# Pan vs. Selective Computed Tomography Scans in Management of Multiple Trauma Patients; a Brief Report 

**Published:** 2017-01-11

**Authors:** Anita Sabzghabaei, Majid Shojaee, Hamid Kariman, Mohammad Manouchehrifar, Kamran Heydari, Sirus Sohrabi

**Affiliations:** 1 Department of Emergency Medicine, Loghmane Hakim Hospital, Shahid Beheshti University of Medical Sciences, Tehran, Iran.; 2 Department of Emergency Medicine, Imam Hossein Hospital, Shahid Beheshti University of Medical Sciences, Tehran, Iran.; 3 Department of Emergency Medicine, Shahid Beheshti Hospital, Qom University of Medical Sciences, Qom, Iran.

**Keywords:** Tomography, x-ray computed, multiple trauma, whole body imaging, emergency service, hospital, diagnostic techniques and procedures

## Abstract

**Introduction:**

Using pan or selective computed tomography (CT) scan in management of multiple trauma patient is a matter of debate. Therefore, the present study was designed aiming to compare the findings of pan and selective CT scans in management of multiple trauma patients.

**Method:**

This is a prospective cross-sectional study, on patients presented to the emergency department (ED) of Shohadaye Haftome Tir Hospital, Tehran, Iran, following blunt multiple trauma over a 1-year period, from March 2014 to March 2015. Findings regarding presence or absence of injury in head, face, neck, chest, abdomen and hip were compared between patients that underwent pan and selective CT using SPSS 21.

**Results:**

443 patients with the mean age of 34.54 ± 17.88 years were evaluated (78% male). 248 (56%) patients underwent selective CT scan and 195 (44%) underwent pan CT scan. The 2 groups were similar regarding vital signs and mean age. Mean hospital length of stay was 21.05 ± 24.64 days for selective CT scan group and 18.18 ± 22.75 days for the other one (p = 0.209). A significant difference was only seen regarding findings of chest injury between the 2 groups (p < 0.001). In other cases a proper overlap was seen between findings of the 2 groups.

**Conclusion:**

Based on the results of the present study, it seems that doing selective CT scan yields results similar to pan CT in detection of head and face, neck and abdomen and hip injuries in multiple trauma patients. However, using pan CT in these patients led to 16% increase in detection and diagnosis of traumatic intra-thoracic injuries.

## Introduction

Modern trauma care puts an emphasis on diagnosis and treatment of injuries in the shortest time possible. Computed tomography (CT) scan is one of the most effective techniques in modern medicine, which is helpful in this regard (1-3). Despite the high capacity of this type of imaging in injury detection, we should note that the new generation of CT scan devices are very expensive and have a high maintenance cost. Therefore, it is very important to do the scan in necessary cases to avoid unnecessary costs and aid in rapid and correct medical decision making. This is even more important in emergency cases, such as trauma patients, where rapid decisions can save a patient’s life (3-6). In cases of multiple-organ trauma, the required scan may be either selective (scan from a pre-determined point) or non-selective (whole body scan from head to hip). Due to its more accurate diagnosis, detection of hidden injuries in asymptomatic cases and aid in more rapid and correct decision making, pan CT scan is very interesting for some physicians (6, 7). However, requesting imaging is accompanied by exposing the patient to a high dose of radiation (7). If the hidden injuries detected in pan CT scan are not clinically significant and do not make a difference in management of the patients, selective CT scan can be used instead, to decrease costs and radiation received and its side effects (7, 8). Nevertheless, some studies do not agree, and believe that selective CT scan is not capable of detecting all injuries caused by blunt trauma (9, 10). Therefore, the present study was designed aiming to compare the findings of pan and selective CT scans in management of multiple trauma patients.

## Methods

***Study design and setting***

This study is a prospective cross-sectional one, with the aim of comparing pan and selective CT scan findings in patients presented to the emergency department (ED) of Shohadaye Haftome Tir Hospital, Tehran, Iran, following blunt multiple trauma over a 1-year period, from March 2014 to March 2015. Non-randomized convenience sampling was used, however since the main researcher’s shifts were well-distributed regarding day or night, and holiday or weekday, patient inclusion was most probably random and unbiased. Based on the protocol of the hospital, both types of imaging were routinely used in management of patients, according to the in-charge physician’s preference. Decisions regarding doing CT scan were usually made based on request from the senior emergency medicine resident and approval of the in-charge surgeon, and the researchers did not interfere with the routine diagnosis and treatment procedures.

All multiple trauma cases caused by falling or traffic accidents, who underwent selective or pan CT scan were included. In cases of selective CT scan, for ruling out the probability of other organ injuries, repeated physical examination, clinical decision rules (11, 12), plain radiography, and ultrasonography were used. In these cases, patients were followed until the final diagnosis regarding the presence or absence of injury in organs that were not scanned, was confirmed. The final decision in this regard was made by the senior emergency medicine resident and the in-charge surgeon.

After completion of diagnostic procedures and reaching a final diagnosis, patient data regarding presence or absence of injury in head, face, neck, chest, abdomen, and hip were gathered for both imaging protocols. To gather data, a checklist was used for each patient that consisted of demographic data (age and sex), hemodynamic status (heart rate, systolic and diastolic blood pressure), level of consciousness based on Glasgow coma scale (GCS), trauma severity based on injury severity score (ISS), hospital length of stay, and final findings regarding presence or absence of injury in head, face, neck, chest, abdomen and hip. Interpretation of the obtained CT scans was done by the senior emergency medicine resident and in-charge surgeon. To increase the confidence, all images were re-interpreted by a radiologist. Disagreements in this regard, were resolved by consulting a third person, either a radiologist or a surgeon or emergency medicine specialist. All CT scans were observed digitally via a computer monitor. To keep personal data and patient information confidential, all researchers adhered to the principles of Helsinki Declaration. Protocol of the present study was approved by the Ethics Committee of Shahid Beheshti University of Medical Sciences. Since the researchers only gathered the data and reports of the routine procedures for patients and did not directly interfere with diagnostic and treatment procedures, no additional cost or delay in treatment was imposed by them.

***Statistical analysis***

Data were analyzed using SPSS version 22. Mean ± standard deviation (SD) was used to report quantitative data, and frequency and percentage were reported for qualitative ones. To compare means between the 2 groups, t-test and ANOVA were employed. P values under 0.05 were considered significant.

## Results

443 patients with the mean age of 34.54 ± 17.88 years (1 – 91) were evaluated (78% male). 248 (56%) patients underwent selective CT scan and 195 (44%) underwent pan CT scan. Table 1 compares baseline characteristics of the patients between the 2 groups. The 2 groups were similar regarding vital signs and mean age. Despite the statistically significant difference between the groups regarding trauma severity and level of consciousness, they were not clinically important. Mean hospital length of stay was 21.05 ± 24.64 days for selective CT scan group and 18.18 ± 22.75 days for the other one (p = 0.209). Table 2 compares the final outcome of selective and pan CT scans regarding traumatic injuries of head, face, neck, chest, abdomen, and hip. A significant difference was only seen regarding findings of chest injury between the 2 groups. In other cases a proper overlap was seen between findings of the 2 groups.

**Table 1:** Baseline characteristics of the studied patients

**Variable **	**Computed tomography scan**		**P value**
	**Selective**	**Pan** 1	
**Age (year)**	34.75 ± 16.91	34.27 ± 18.64	0.781
**Trauma severity** *	21.96 ± 12.36	24.74 ± 13.20	0.023
**Heart rate (beats/minute)**	94.48 ± 18.50	95.65 ± 21.87	0.540
**Systolic BP (mmHg)**	118.40 ± 23.48	118.53 ± 22.74	0.955
**Diastolic BP (mmHg)**	75.16 ± 12.28	74.33 ± 12.68	0.489
**Level of consciousness** #	12.6 ± 3.66	11.34 ± 4.04	0.052

1 Pan: whole body scan from head to hip,

* based on injury severity score (ISS), BP: blood pressure,

# based on Glasgow coma scale. Measures are reported as mean ± standard deviation.

**Table 2:** Comparison of final findings in selective and pan computed tomography (CT) scan groups regarding anatomic site of injury

**Anatomic site of injury**	**CT scan; n (%)**		**P value**
	**Selective**	**Pan** 1	
**Head**			
Yes	124 (54.6)	103 (45.4)	0.311
No	124 (57.4)	92 (42.6)	
**Face**			
Yes	61 (57.5)	45 (42.5)	0.738
No	187 (56)	149 (44)	
**Neck**			
Yes	13 (48.1)	15 (51.9)	0.363
No	235 (56.6)	180 (43.3)	
**Chest**			
Yes	63 (42)	87 (58)	< 0.001
No	185 (63.4)	108 (36.6)	
**Abdomen and hip**			
Yes	63 (53.4)	55 (46.6)	0.588
No	140 (43.2)	184 (56.8)	

1 Pan: whole body scan from head to hip.

## Discussion

Based on the findings of the present study, patients who underwent selective and pan CT scan were in a similar state regarding vital signs, level of consciousness, trauma severity and mean age and the final findings of the patients regarding head and face, neck, abdomen and hip were not significantly different. Only in thoracic injuries, the rate of pathologic findings was significantly higher in the group that underwent pan CT scan (58% vs. 42% in selective CT scan). Sufficient data is not available regarding the types of detected findings; however, the difference in thoracic findings might be due to detection of hidden hemothorax cases via pan CT scan.

Advances in imaging technology have been very helpful in more rapid and accurate diagnosis in recent years. Using these techniques has grown in EDs since it decreases the time needed for reaching a diagnosis (13-17). Using spiral CT scan has reduced the patients’ ED length of stay from 85 minutes to 45 minutes (18). Although using imaging techniques has significantly improved management of trauma patients, their protocol of use is a matter of debate due to the side effects and financial burdens (14, 15, 19). In America, it has been estimated that two third of the radiation received from imaging is from CT scan and this has increased the risk of mortality from radiation to 12.5 cases in 10000 CT scanned population (20). Currently, there is no consensus regarding definite indications of using CT scan in management of trauma patients and utilization of this kind of imaging largely depends on the opinion of the in-charge physician. Although pan scan imposes a high dose of radiation on the patient, sometimes correct diagnosis of injury in multiple trauma patients and saving their life is more important than the dose of radiation received.

Based on the results of a study, more than 50% of trauma patients with normal chest radiography showed evidence of a traumatic chest injury when underwent CT scan (however, only 8% of these injuries were clinically important). This study has strongly recommended doing CT scan for all patients with severe chest injuries (1). At the same time, non-selective CT scan has decreased waiting time, from ED arrival to receiving emergency care, for patients with severe multiple trauma (21). Pan CT scan is more rapid and has higher quality in diagnosis of injuries to different parts of the body. Yet, due to the high dose of radiation and expenses imposed on the patient, doing pan CT for all trauma patients is still a matter of debate. Wagner et al. introduced non-selective scan as a standard method for evaluating multiple trauma patients and Caputo et al. have deemed it a desirable method in managing these patients (8, 22). On the other hand, in a study by Gupta et al. doing selective CT scan led to a decrease in scan frequency and few undiagnosed injuries were highly important (7). In addition, in a study by Deunk et al. selective abdominal and chest scan helped a lot in making a decision in 34% of blunt trauma patients (23).

Based on the results of the present study, selective and pan CT scan have similar value in diagnosis of injuries in different parts of the body in trauma patients. However, pan CT scan led to a 16% increase in detection of chest trauma injuries, which is in line with the results of a similar study in this field (1).

Not recording the types of injuries detected and their importance in the patient’s final outcome is among the limitations of the present study, since presence of a finding such as occult pneumothorax does not make a difference in management of the patient if they are not in need of mechanical ventilation. Therefore, it seems that to reach a solid decision regarding cost and benefit of these CT scan methods for trauma patients more accurate studies and analytical evaluation of the severity of the injuries not diagnosed in selective scan are needed. 

## Conclusion:

Based on the results of the present study, it seems that doing selective CT scan yields results similar to pan CT in detection of head and face, neck and abdomen and hip injuries in multiple trauma patients. However, using pan CT in these patients led to 16% increase in detection and diagnosis of traumatic intra-thoracic injuries. 
